# A rare case of metastasized non-functional pancreatic neuroendocrine tumor with a good long-term survival

**Published:** 2016

**Authors:** A Mirică, IA Bădărău, R Mirică, S Păun, DL Păun

**Affiliations:** * ”C. I. Parhon” National Institute of Endocrinology, Bucharest, Romania; ** Department of Physiology I, “Carol Davila” University of Medicine and Pharmacy, Bucharest, Romania; *** ”Sf. Ioan” Emergency Hospital, Bucharest, Romania; **** ”Floreasca” Clinical Emergency Hospital, Bucharest, Romania

**Keywords:** pancreatic non-functional well-differentiated neuroendocrine tumor, survival rate, type 2 diabetes

## Abstract

**Background:** Non-functional neuroendocrine tumors of the pancreas (NF-pNETs) are a varied group of extremely rare malignancies. The majority of patients already have liver metastases at the diagnosis moment, thus, treatment options are restricted, and the survival rate is reserved.

**Case report:** We presented the case of 59-year-old patient, diagnosed with non-functional well-differentiated pancreatic neuroendocrine tumor grade II (NET G2) with the presence of chromogranin A, synaptophysin and somatostatin receptor 2, together with liver and bone metastases. Patient underwent a surgical excision of the pancreatic tumor, started long-acting somatostatin analogues (octreotide), interferon therapy for liver metastases and local radiotherapy for bone metastases. After one year, the patient developed diabetes, needing insulin therapy. At approximately three years after the diagnosis, the patient was still living, had a good quality of life, and was free of local recurrence of the tumor or other metastases.

**Conclusion:** Our case report presented a rare case of metastatic non-functional well-differentiated pancreatic neuroendocrine tumor, involving a multidisciplinary therapeutic approach in order to obtain a good long-term survival.

## Introduction

Pancreatic neuroendocrine tumors (p-NETs) are a captivating and diverse group of rare neoplasms, which arise from pancreatic islet cells. In this article, we presented the case of a female patient with non-functional pancreatic neuroendocrine tumor with multiple liver and bone metastases, whose therapy included a multidisciplinary team of medical specialties: surgery, endocrinology, oncology, radiology, and gastroenterology. After treatment, the metastases were monitored, with no local recurrence of primary tumor. One year after the excision of the pancreatic head and tail, the patient developed diabetes, needing insulin therapy.

## Case report

**Fig. 1:**
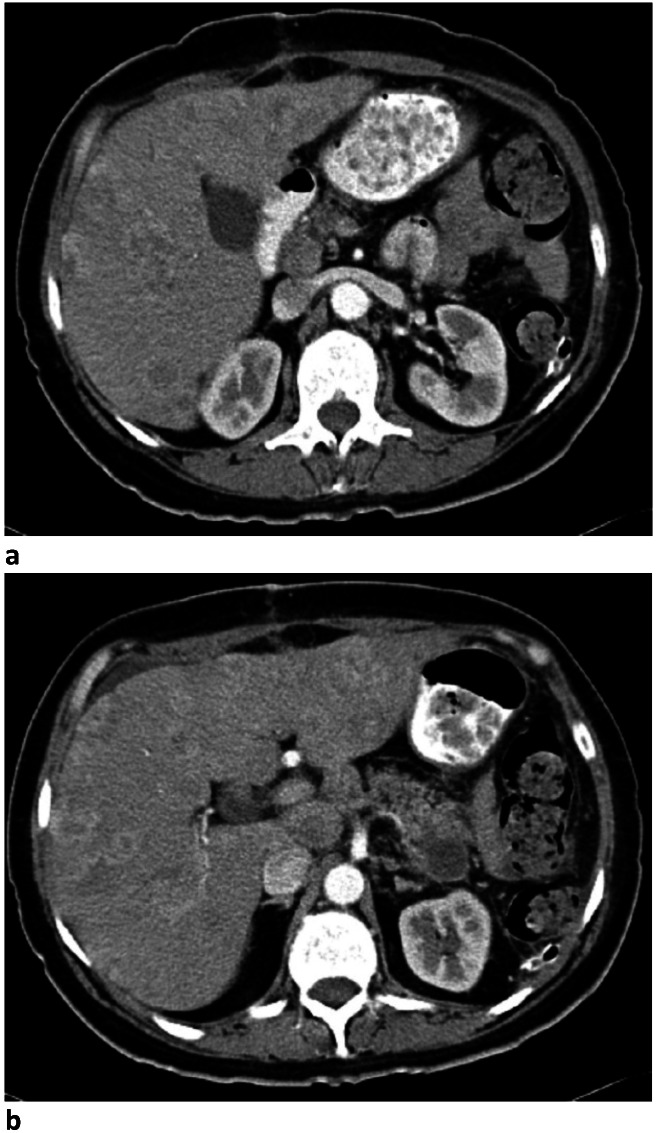
Computed tomography location and aspects of pancreatic tumor a,b

In 2013, a 59-year-old white Caucasian non-smoking woman presented to the surgery department describing symptoms of diffuse abdominal pain, weight loss (18 kilograms/1 year) and unselective anorexia with the insidious onset of symptoms in the previous 6 months. The patient reported a personal history of chronic hepatitis C treated with pegylated interferon and ribavirin 16 years before and no particular familial history.Laboratory tests were within normal ranges, except for an elevated alkaline phosphatase of 158 u/l (normal range <104 U/L), a moderate hepatic cytolysis with a small elevation of transaminases, alanine transaminase (ALT) of 36 U/L (normal range 5-21 U/L) and aspartate transaminase (AST) of 37 U/L (normal range 5-30 U/L ) and increased carcinoembryonic antigen of 9.75 ng/mL (normal range for non-smokers < 3 ng/ mL). Pelvic and abdominal computer tomography (CT) with contrast substance revealed hepatomegaly with multiple hypodense formations, the largest with a diameter of 3 cm and a pancreatic tumor of 4/ 3.8 cm diameter, with distal development, involving hilum splenic (**Fig. 1 a,b**).

**Fig. 2:**
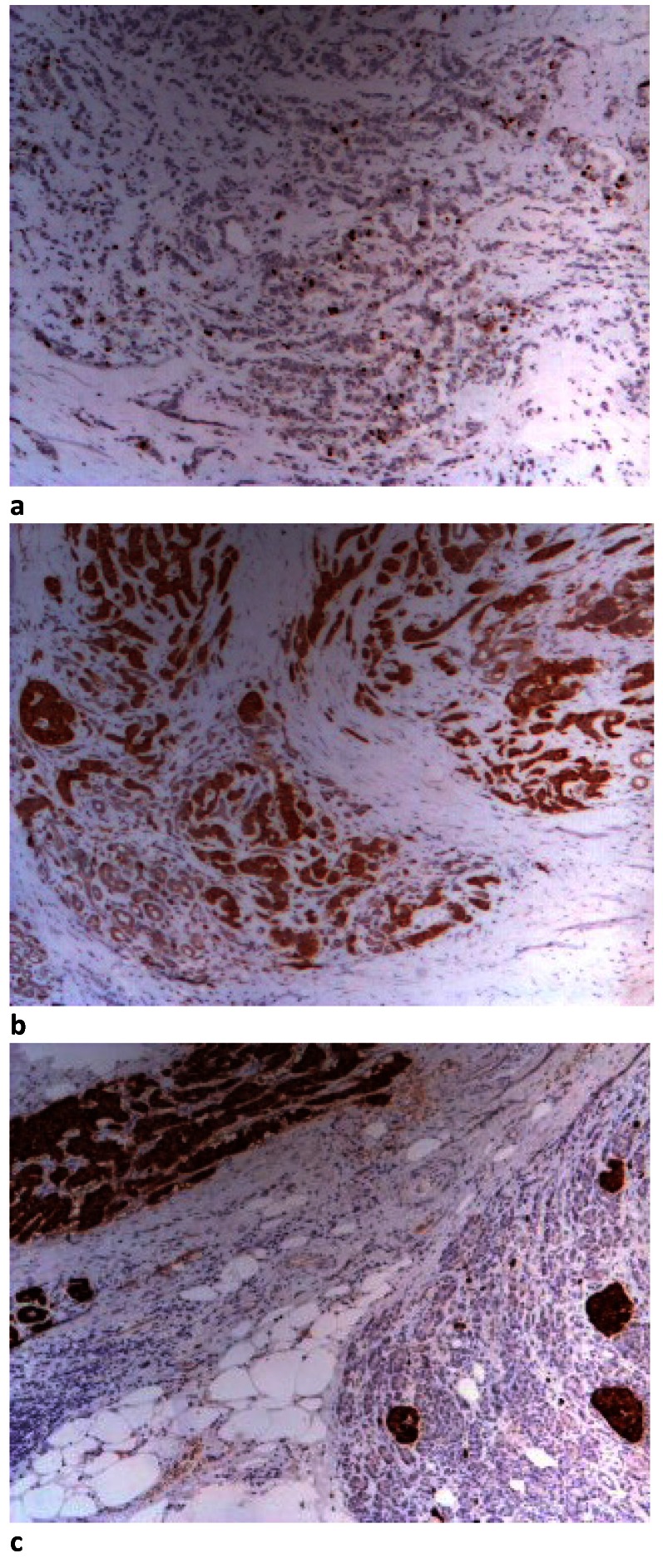
Immunohistochemistry aspects of neuroendocrine pancreatic tumor a. Diffuse positive marker Ki67 15-20% in tumor cells, b. b. Diffuse positive synaptophysin in tumor cells, 4X optical microscopy, c. Diffuse positive chromogranin A in tumor cells

A surgical laparoscopic intervention, which revealed numerous whitish tumor formations of 5-30 mm at both liver lobes, without peritoneal carcinomatosis, was performed. A conversion to median celiotomy was decided, finding a 4/4 cm pancreatic tumor located in the distal part of the pancreas, involving peripancreatic tissues and the vessels of splenic hilum. Then, a distal pancreatectomy and splenectomy was performed. Postoperative CT scan performed at 2 weeks after surgery revealed the presence of a pseudocystic collection in the pancreatic stump, of 3.8/ 3.4 cm, which was effectively drained through the locally installed drain. Histopathological and immunohistochemical results diagnosed well-differentiated pancreatic neuroendocrine tumor grade II (NETG2) with liver metastases and no spleen invasion, with the presence of diffuse positive chromogranin A and synaptophysin. The cellular marker for proliferation Ki67 was 15 to 20% and the tumor cells also expressed somatostatin receptor 2 (SSR2) (**Fig. 2 a,b,c**). 

The patient was subsequently sent to the endocrinology service, where extensive further medical tests were conducted. Hormonal investigations indicated an elevated serum chromogranin A of 468 ng/ mL (normal range 20-125 ng/mL) and serum neuron specific enolase of 56 ng/mL (normal range 0-12 ng/mL) with normal values of plasmatic serotonin and urinary 5-hydroxyindoleacetic acid. Furthermore, endocrine investigations including pituitary, thyroid, parathyroid, and adrenal hormones were normal and even though genetic testing was not possible, MEN1 syndrome was excluded. Moreover, a bone scintigraphy with technetium 99 was performed and it revealed multiple bone metastases in the vertebral body L3, L4, coccygeal region, posterior rib 10 and 6, left iliac wing, right clavicle and left femoral diaphysis. Based on these results, after a multidisciplinary cooperation between the oncologist, endocrinologist and radiotherapist, the patient started treatment with long-acting somatostatin analogues (octreotide) 30 mg at 4 weeks, interferon alfa 2B – 3 million units three times a week for 6 months and a single local external radiation therapy (dose of 8 Gy) for the relieve of lumbar pains. After completing treatment with interferon, the patient began systemic chemotherapy with 5-fluorouracil. The patient was monitored at short intervals, with no local recurrence of the pancreatic tumor or other metastases and the optimal control of symptoms was obtained. The follow-up examination, one year later, revealed elevated glycemic values, diagnosed as a type 2 diabetes and the patient was started on therapy with long-acting insulin.

## Discussions

P-NETshave a low incidence of less than 1 per 100,000 persons, representing 1.3-2% of all types of pancreatic tumors.They are classified as functional p-NETs, associated with a particular clinical syndrome such as Cushing syndrome and non-functional p-NETs, frequently asymptomatic, associated with no particular medical sign. Non-functional p-NETs frequently secrete chromogranin A, neuron-specific enolase, human chorionic gonadotrophin subunits, pancreatic polypeptide or other peptides, but they do not manifest with particular clinical symptoms and thus they are referred as non-functional tumors. At the same time, approximately 10–30% of pNETs are functional and the most common functional p-NETs are gastrinomas and insulinomas, with the rare functional p-NETS represented by vasoactive intestinal peptide tumor (VIPomas) and somatostatinomas [**1**,**2**]. Most p-NETs are sporadic, but in 10% of cases they can occur in association with the hereditary syndromes such as MEN-1 syndrome, Von Hippel Lindau syndrome, tuberous sclerosis and neurofibromatosis type 1 [**3**,**4**]. 

Non-functional p-NETS frequently present with distant metastasis, especially to the liver and bones, as in our patient’s case, due to their asymptomatic nature. Although the majority of p-NETs occur in the head of the pancreas, in our patient’s case, the tumor affected the body and the tail of the pancreas [**5**,**6**]. 

The diagnostic strategy includes localization of the tumor (abdominal CT or MRI, somatostatin receptor scintigraphy) and laboratory tests, in order to confirm the functional or non-functional status. Therapeutic management includes surgery as first choice therapy in resectable tumors, somatostatin analogue or specific molecular targeted therapy,systemic chemotherapy and liver-directed therapies (radiofrequency ablation, hepatic artery embolization) in patients with a metastatic disease [**7**,**8**]. In our patient’s case, the first choice of treatment was the surgical excision of the pancreatic tumor, followed by treatment with somatostatin analogues and medical therapy for liver metastases, noting that the patient had diffuse bilobed involvement with associated bone metastases. In addition, systemic chemotherapy and a single dose of radiation therapy have been recommended. Furthermore, in our case, the occurrence of diabetes might have been either due tothe partial excision of the pancreas or due to the treatment with somatostatin analogues, which are known to reduce theinsulin levels secreted by the pancreatic cells. 

Patients with p-NETS with metastatic disease have a worse prognosis, with a median survival rate of 22 months [**9**,**10**]. Our patient is still living, having a good quality of life of approximately 34 months since the diagnosis moment. 
